# Gastric SMARCA4-deficient undifferentiated tumor (SMARCA4-UT): a clinicopathological analysis of four rare cases

**DOI:** 10.1186/s13023-024-03244-4

**Published:** 2024-06-14

**Authors:** Ping Zhou, Yiyun Fu, Weiya Wang, Yuan Tang, Lili Jiang

**Affiliations:** Department of Pathology, West China Hospital, Sichuan University, No. 37 Guo Xue Xiang, Chengdu, 610041 Sichuan P.R. China

**Keywords:** SWI/SNF, SMARCA4, BRG1, Gastric cancer, Undifferentiated tumor

## Abstract

**Background:**

SMARCA4, as one of the subunits of the SWI/SNF chromatin remodeling complex, drives SMARCA4-deficient tumors. Gastric SMARCA4-deficient tumors may include gastric SMARCA4-deficient carcinoma and gastric SMARCA4-deficient undifferentiated tumor (SMARCA4-UT). Gastric SMARCA4-UT is rare and challenging to diagnose in clinical practice. The present report aims to provide insight into the clinicopathological characteristics and genetic alterations of gastric SMARCA4-UTs.

**Results:**

We retrospectively reported four rare cases of gastric SMARCA4-UTs. All four cases were male, aged between 61 and 82 years. These tumors presented as ulcerated and transmural masses with infiltration, staged as TNM IV in cases 1, 2 and 4, and TNM IIIA in case 3. Pathologically, four cases presented solid architecture with undifferentiated morphology. Cases 2 and 3 showed focal necrosis and focal rhabdoid morphology. Immunohistochemical staining showed negative expression of epithelial markers and deficient expression of SMARCA4. Furthermore, positivity for Syn (cases 1, 2 and 3) and SALL4 (cases 1 and 2) were observed. Mutant p53 expression occurred in four cases, resulting in strong and diffuse staining of p53 expression in cases 1, 2 and 4, and complete loss in case 3. The Ki67 proliferative index exceeded 80%. 25% (1/4, case 4) of cases had mismatch repair deficiency (dMMR). Two available cases (cases 1 and 3) were detected with *SMRACA4* gene alterations. The response to neoadjuvant therapy was ineffective in case 1.

**Conclusions:**

Gastric SMARCA4-UT is a rare entity of gastric cancer with a poor prognosis, predominantly occurs in male patients. The tumors are typically diagnosed at advanced stages and shows a solid architecture with undifferentiated morphology. Negative expression of epithelial markers and complete loss of SMARCA4 immunoexpression are emerging as a useful diagnostic tool for rare gastric SMARCA4-UTs.

## Background

Gastric cancer (GC) is one of the most common malignancies globally and has poor outcome, especially in Asia [1, 2]. According to the fifth edition of the World Health Organization (WHO) classification of digestive tumors, undifferentiated gastric carcinomas are rare highly aggressive tumors showing no specific cytological or architectural type of differentiation [3]. Some undifferentiated gastric carcinomas may exhibit rhabdoid features, comprising 0.1–0.3% of poorly differentiated adenocarcinoma, and 5.0-5.6% of solid adenocarcinomas as reported in literature [4, 5]. The undifferentiated phenotype is probably driven by various components of the switch/sucrose non-fermenting (SWI/SNF) chromatin-remodeling complex in some cases [3, 6, 7].

Kadoch et al. demonstrated that mutations in SWI/SNF chromation-remodeling complex are present in approximately 20% of all human cancers [8]. SWI/SNF complex is a highly preserved group of multiprotein complexes responsible for regulating chromatin remodeling and play a crucial role in proliferation, differentiation and tumor suppression [9, 10]. Recent studies have reported that the undifferentiated phenotype is probably driven by various components of the SWI/SNF complex [6, 11–14]. SMARCA4, also known as BRG1, is the ATPase subunit of the SWI/SNF complex. *SMARCA4*, a tumor suppressor located on chromosome 19p13.2, is aberrant in approximately 5–7% of all human malignancies [15]. *SMARCA4* mutations were interpreted as pathogenic in 3.6% (42/1174) of gastroesophageal carcinomas [16]. SMARCA4 (BRG1) deficiency has been reported in several malignances [17–24], including small cell carcinoma of the ovary, hypercalcemic type (SCCOHT), lung adenocarcinoma, medulloblastoma, Burkitt lymphoma, breast cancer, uterine sarcoma, and thoracic SMARCA4-deficient undifferentiated tumors (SMARCA4-UT).

Immunohistochemical staining for SMARCA4 is useful to identify SMARCA4-deficient tumors. Gastric SMARCA4-deficient tumor may include gastric SMARCA4-deficient carcinoma and gastric SMARCA4-UT. Gastric SMARCA4-deficient carcinoma could be differentiated from gastric SMARCA4-UT based on gland architecture, cellular cohesion and diffuse strong keratin expression. Furthermore, gastric SMARCA4-UT is typical loss of epithelial differentiation (negative expression of epithelial markers such as pancytokeratin (PCK) and epithelial membrane antigen (EMA)), whereas gastric SMARCA4-deficient undifferentiated carcinoma reveals variable expression of epithelial markers [13].

Gastric SMARCA4-UT is a rare entity of gastric tumors. Due to its rarity, the clinicopathological significance and molecular features are limited. Herein, we present four rare cases of gastric SMARCA4-UTs and provide insight into the clinicopathological characteristics and genetic alterations of these highly aggressive malignant tumors.

## Results

### Clinical features

Four cases diagnosed with gastric SMARCA4-UTs were all male, aged between 61 and 82 years. These tumors presented with ulcerated and advanced tumors, staged as tumor-node-metastasis (TNM) IV in cases 1, 2 and 4, and TNM IIIA in case 3. Tumor size varied from 2 to 6 cm in maximum diameter. Detailed clinical features are summarized in Table 1.

**Table 1:** The clinicopathological characteristics of gastric SMARCA4-UTs

Characteristics	Case 1	Case 2	Case 3		Case 4
**Clinical features**					
Age/Gender	61/M	64/M	61/M		82/M
Symptoms	Dysphagia, abdominal pain and bloating	Intermittent hematemesis, abdominal pain	Upper abdominal discomfort with bloating		Abdominal pain
Tumor size	6 cm	2 cm	4 cm		3 cm
Tumor location	The cardia, fundus, and lesser curvature of the stomach	Lower esophagus, cardia of the stomach	Lateral posterior wall of the stomach		Antrum of the stomach
TNM Stage	IV	IV	IIIA		IV
**Histological features**					
Pattern	Solid architecture	Solid architecture	Solid architecture		Solid architecture
Necrosis	-	Focal necrosis	Necrosis		-
Rhabdoid features	-	Focal rhabdoid features	Focal rhabdoid features	-	
**IHC features**					
SMRACA4 (BRG1)	Complete loss	Complete loss	Complete loss		Complete loss
SMARCB1 (INI1)	Retained expression	Retained expression	Retained expression		ND
PCK	-	-	-		-
EMA	-	-	-		-
CK7	-	-	-		-
SALL4	+	+	ND		ND
CD34	-	ND	+		ND
Syn	+	+	+		ND
CgA	-	-	-		ND
CD56	-	-	-		ND
P53	+	+	-		+
Ki67	80%	90%	80%		80%
MLH1	+	+	+		-
MSH2	+	+	+		+
MSH6	+	+	+		+
PMS2	+	+	+		-
Others (negative)	CK5/6, NUT, CDX2, CD117, CD20, CD5, CD3, CD79a, CD30, S100, LCA, HMB45, MUM1, CD43, MPO, TDT and p63	CK5/6, CK20, Myogenin, MyoD1, Desmin, CEA, S100, CK8/18, CDX2, CD20, CD5, CD3, CD79a, LCA and HMB45	CK8/18, CK20, CAM5.2, S100, HMB45, CD117, DOG1, CD99, WTI, Desmin and SMA		LCA
**EBER**	-	-	ND		ND
***SMARCA4*** **gene alteration**	Deletion mutation of *SMARCA4*	ND	Translocation of *SMARCA4* and *LDLR*		ND
**Treatment**	Biopsy + neoadjuvant therapy + surgical resection + chemotherapy	Biopsy + chemotherapy	Surgical resection + chemotherapy		Biopsy
**MTS/Survival**	Liver metastasis AWD (17 months)	Liver metastasis AWD (4 months)	NA		Peritoneal metastases AWD (4 months)

M, male; y, years; TNM, tumor-node-metastasis; IHC, immunohistochemistry; +, positive; -, negative; MTS, metastasis. AWD, alive with disease; NA, not available; ND, not done

**Fig. 1:**
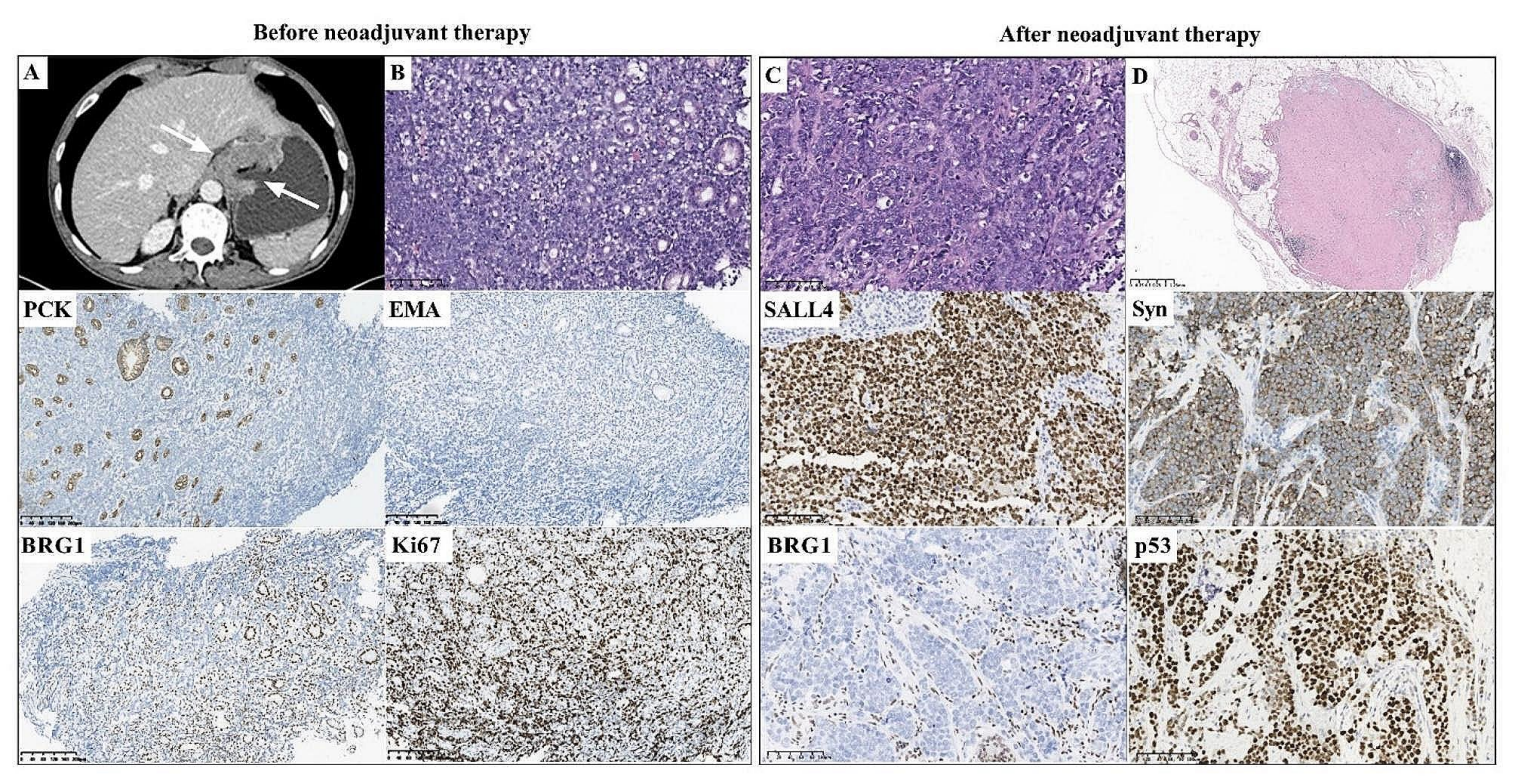
Radiological, histopathological and immunohistochemical characteristics of case 1. **A** CT scan of the abdomen showed a large irregular thickened area of the cardia, fundus and lesser curvature of the stomach before neoadjuvant therapy(**A**). The tumor showed a solid architecture with undifferentiated morphology before (**B**, magnification x200) and after neoadjuvant therapy (**C**, magnification x200). There was no obvious response to neoadjuvant therapy (TRG3) (**C**, magnification x200). However, the lymph nodes showed a response to neoadjuvant therapy (**D**, magnification x10). Both biopsy and surgical resection specimen showed similar immunohistochemical staining. BRG1 was deficient expression in the nucleus of tumor cells. Both PCK and EMA were negative expression. The Ki67 proliferation index was approximately 80%. SALL4 and Syn were positively stained. P53 was positively expressed. (Magnification x200)

A 61-year-old male patient (case 1) who presented with dysphagia, abdominal pain and bloating for approximately one month was admitted to our hospital. A computed tomography (CT) scan of the abdomen showed a 6 cm irregular thickened tumor in the cardia, fundus and lesser curvature of the stomach (Fig. 1A). There were multiple enlarged lymph nodes in the ligamenta hepatogastricum, portacaval space, mesentery and abdominal aorta, which were partially fused, and a nodule in right posterior lobe of the liver. Gastroscopy showed a large ulcerative tumor in the cardia of the stomach, and a biopsy was performed. The clinical TNM stage was TNM VI. The patient received neoadjuvant therapy with a combination of chemotherapy (irinotecan and cisplatin) and sintilimab (a PD-1 inhibitor). A partial response (PR) was achieved after two cycles. The patient received continued treatment after six cycles with progressive disease (PD). Subsequently, this patient received the second line chemotherapy with albumin paclitaxel combined with tigio, and a PR was achieved after three cycles in clinical assessment. And then, surgical resection was performed.

**Fig. 2:**
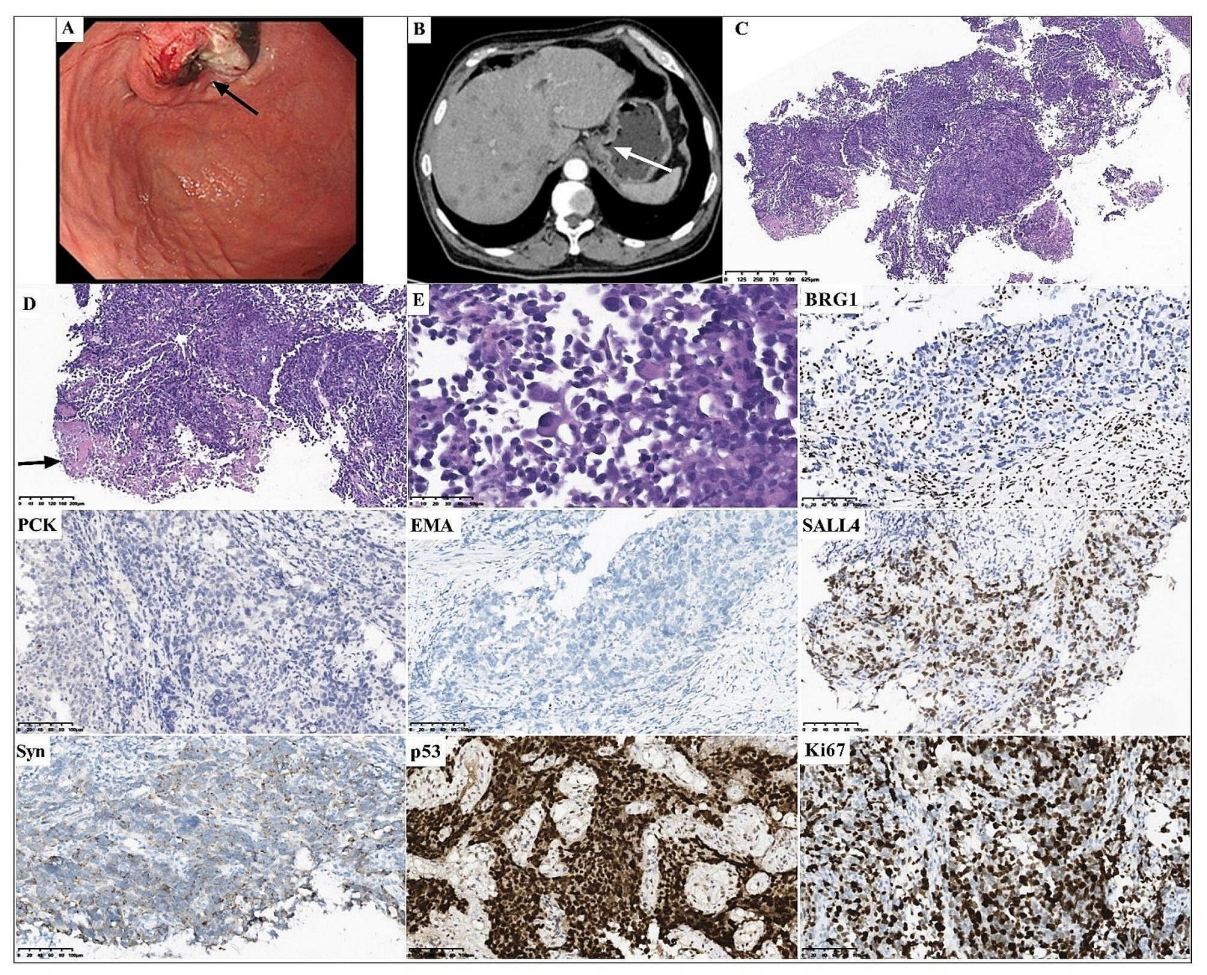
Gastroscopic, radiological, histopathological and immunohistochemical characteristics of case 2. Gastroscopy (**A**, arrow) and CT scan (**B**, arrow) showed a thickened lesion in lower esophagus and cardia of the stomach. The tumor showed a solid architecture with undifferentiated morphology (**C**, magnification x40) with focal necrosis (**D**, arrow, magnification x200). Partial tumor cells showed a rhabdoid morphology (**E**, magnification x200). BRG1 was deficient expression. Immunohistochemical staining showed negativity for both PCK and EMA, diffuse positivity for SALL4, and partial positivity for Syn. P53 was positively expressed. The Ki67 proliferation index was approximately 90%. (Magnification x200)

A 64-year-old male patient (case 2) presented with intermittent hematemesis and abdominal pain for three months. The CT scan of the abdomen and gastroscopy showed a 2 cm thickened lesion in lower esophagus and cardia of the stomach (Fig. 2A-B). A biopsy was performed. Besides, the CT scan showed multiple nodules in liver, indicating metastases of the liver; Additionally, lymph nodes in hepatogastric ligaments and portal space were enlarged and partially fused. The clinical TNM stage was TNM VI.

A 61-year-old male patient (case 3) presented with upper abdominal discomfort with bloating for approximately one month and was admitted to the local hospital. Gastroscopy was performed and showed a 4 cm mass in the lateral posterior wall of the stomach. Gastrectomy was performed at the local hospital. The pathological TNM stage was T4aN1M0, IIIA. The patient received chemotherapy after surgical resection.

An 82-year-old male patient (case 4) presented with abdominal pain for approximately one month and was admitted to the local hospital. A CT scan of the abdomen showed a 3 cm thickened and transmural mass in the antrum of the stomach, and metastatic nodules in the adjacent omentum and peritoneum. Multiple lymph nodes in the abdominal cavity and retroperitoneum were enlarged and fused. Gastroscopy was performed at the local hospital and a biopsy was performed. The clinical TNM stage was TNM VI.

### Pathological features

**Fig. 3:**
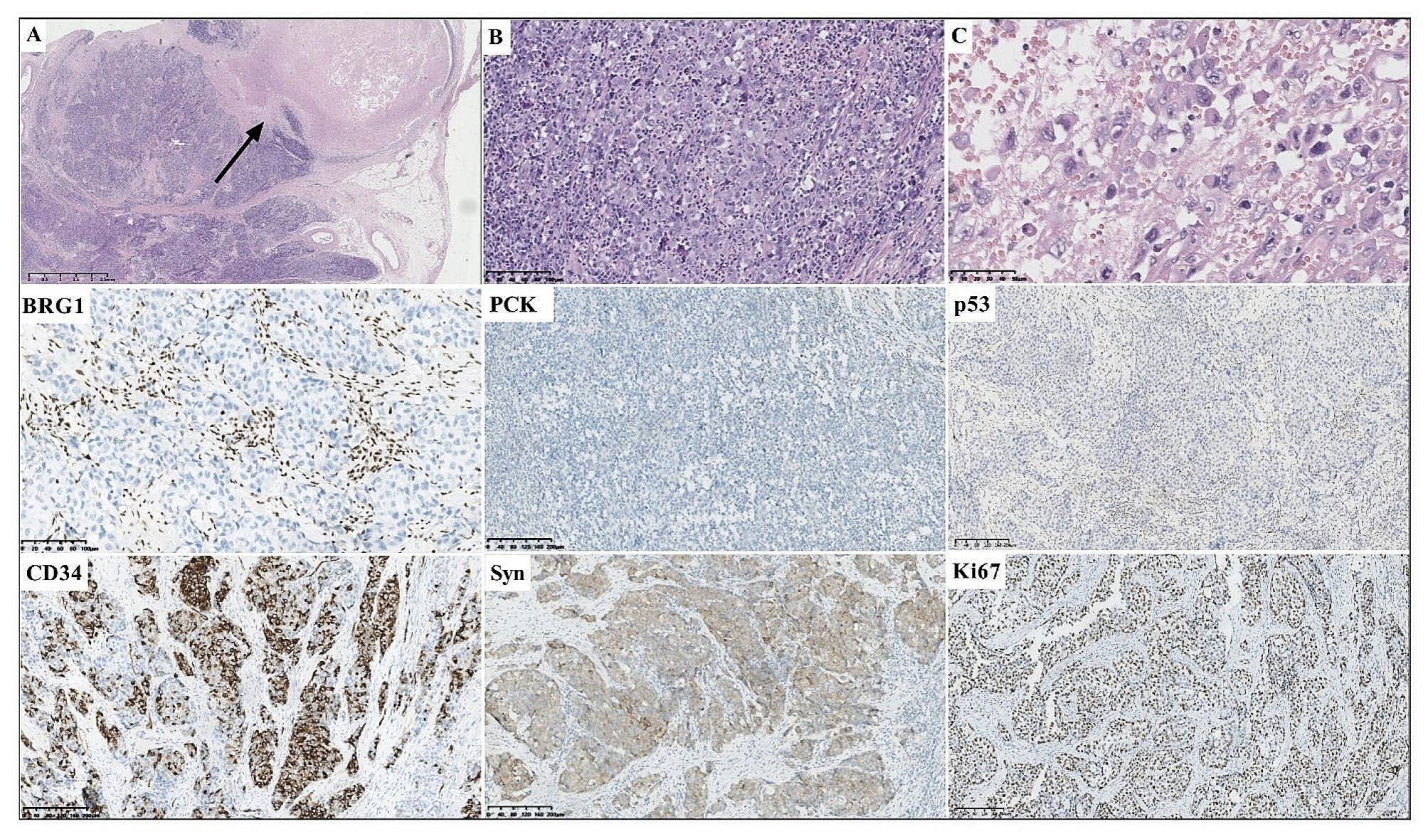
Histopathological and immunohistochemical characteristics of case 3. The tumor showed a solid architecture with comedonecrosis (**A**, arrow). High-power view showed undifferentiated tumor cells with poor cohesion (**B**, magnification x200). Partial tumor cells showed a rhabdoid morphology (**C**, magnification x400). BRG1 was deficient expression. PCK was negative expression. Expression of p53 was completely loss. CD34 and Syn were positively stained. The Ki67 proliferation index was approximately 80%. (Magnification x200)

**Fig. 4:**
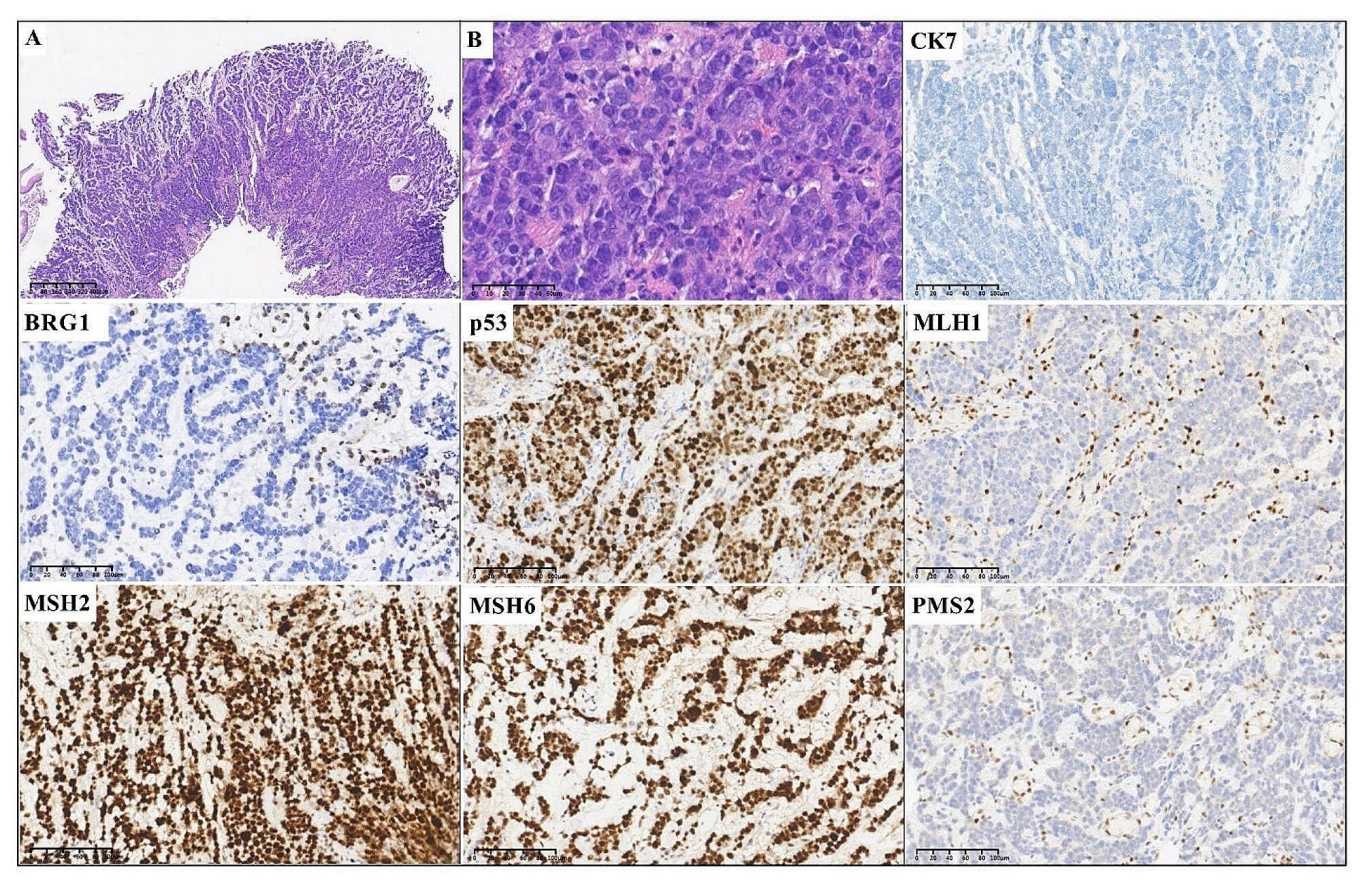
Histopathological and immunohistochemical characteristics of case 4. A low-power view (**A**, magnification x40) and high-power (**B**, magnification x400) of the tumor showed a solid pattern with undifferentiated morphology. Immunohistochemical staining showed negative expression of CK7, deficient expression of BRG1 and positive expression of p53. This case showed mismatch repair deficiency (dMMR), with deficient expression of MLH1 and PMS2, and retained expression of MSH2 and MSH6 (Magnification x200)

Cases 3 and 4 were admitted and treated at the local hospital, and pathological consultation was submitted to our hospital. All four cases had similar histological characteristics. Morphological observation showed undifferentiated tumor cells that formed a solid architecture without tubular glandular formation (Figs. 1B-C, 2C-E and 3A-B, and 4A-B). The large- to medium-sized tumor cells were epithelioid ovoid or polygonal cells with abundant cytoplasm. Round, pleomorphic nuclei with prominent nucleoli were large and irregular. Mitoses were frequent. Cases 2 and 3 showed focal necrosis (Figs. 2D and 3A) and focal rhabdoid morphology (Figs. 2E and 3C).

The tumor regression grade (TRG) for case 1 with neoadjuvant therapy was TRG3 (without an obvious response to neoadjuvant treatment) (Fig. 1C), while the lymph nodes showed a response to neoadjuvant therapy (Fig. 1D).

All four specimens showed complete loss of SMARCA4 (BRG1) in the tumor nuclei, with endothelial and inflammatory cells as internal positive controls (Figs. 1, 2, 3 and 4), and showed negative expression of epithelial markers, including PCK, EMA and/or CK7. Immunohistochemical staining showed SMARCB1 (INI1) retained expression (3/3, cases 1, 2 and 3), partial positivity for Synaptophysin (Syn) (3/3, cases 1, 2 and 3) and positivity for spalt-like transcription factor 4 (SALL4) (2/2, cases 1 and 2) in available cases. Mutant p53 expression occurred in four cases, resulting in strong and diffuse staining in cases 1, 2 and 4, and complete loss of p53 expression in case 3. The Ki67 proliferative index exceeded 80%. 25% (1/4, case 4) of cases had mismatch repair deficiency (dMMR).

The tumor cells of case 1 showed negative staining for PCK, EMA and CK7, diffuse positivity for SALL4 and Syn (Fig. 1). The tumor cells were negative expression of CD34, chromogranin A (CgA), CD56, CK5/6, nuclear protein in testis (NUT), CDX2, CD117, CD20, CD5, CD3, CD79a, CD30, S100, LCA, HMB45, MUM1, CD43, MPO, TDT and p63. MLH1, MSH2, MSH6 and PMS2 were retained expression. The expression of p53 was strong and diffuse staining, and the Ki67 proliferative index was approximately 80%. The immunohistochemistry staining of the resected specimen was similar to that of the biopsy.

The tumor cells of case 2 was negative expression of PCK, EMA and CK7, diffuse positivity for SALL4 and partial positivity for Syn (Fig. 2). The tumor cells were negative expression of CgA, CD56, CK5/6, CK20, Myogenin, MyoD1, Desmin, CEA, S100, CK8/18, CDX2, CD20, CD5, CD3, CD79a, LCA and HMB45. MLH1, MSH2, MSH6 and PMS2 were retained expression. The expression of p53 was strong and diffuse staining, and the Ki67 proliferative index was approximately 90%.

The tumor cells of case 3 was diffusely positivity for CD34 and Syn (Fig. 3). The tumor cells were negative expression of epithelial markers, including PCK, EMA, CK8/18, CK20 and low molecular weight keratin (CAM5.2), as well as CgA, CD56, S100, HMB45, CD117, DOG1, CD99, WTI, Desmin and SMA. MLH1, MSH2, MSH6 and PMS2 were retained expression. The expression of p53 was complete loss, and the Ki67 proliferative index was approximately 80%.

The tumor cells of case 4 was negative expression of PCK, EMA and CK7. The present patient had mismatch repair deficiency (dMMR), with deficient expression of MLH1 and PMS2, and retained expression of MSH2 and MSH6 (Fig. 4). The expression of p53 was strong and diffuse staining, and the Ki67 proliferative index was approximately 80%.

### Molecular analysis

In situ hybridization for EBER was negative in available cases (cases 1 and 2). The next-generation sequencing (NGS) of case 1 confirmed deletion mutations in *SMARCA4, RAD51* and *TSC2*, and the tumor mutation burden (TMB) was 0.96 mutations/Mb. The NGS of case 3 confirmed that SV (structural variation) of *SMARCA4* was caused by translocation of *SMARCA4* and *LDLR*, and *CDH4* was caused by translocation of *CHD4* and *NCAPD2*; point mutation of *KDR, AR, FBXW7, TP53, EP300* and *APC*; deletion mutation of *FBXW7* and *FANCA*; The TMB was 5.3 mutations/Mb.

## Discussion

SMARCA4 is associated with progression and poor prognosis in gastric cancer [7, 25]. Inactivation of SMARCA4 rarely occurs in classic glandular gastric cancer as a driver molecular event and is more likely to occur in gastric cancer with solid and poorly differentiated and undifferentiated morphology [6, 13, 16, 26], and loss of SMARCA4 is associated with adverse clinical characteristics [11, 12]. Patients with undifferentiated carcinoma of the gastrointestinal (GI) tract exhibiting loss of SMARCA4 expression demonstrated significantly poorer overall survival (*p* = 0.028) and disease-free survival (*p* = 0.006) compared to those with SMARCA4 expression [7]. In the present report, all four cases were male aged ranged from 61 to 82 years, presented as large ulcerated and transmural masses with infiltration and were staged as TNM IV in cases 1, 2 and 4, and TNM IIIA in case 3 at the time of diagnosis. Tumors presented solid architecture with undifferentiated morphology and showed complete loss of SMARCA4 (BRG1) expression. In addition, 50% (2/4, cases 2 and 3) showed focal necrosis and rhabdoid morphology, comprised less than 1% of the tumor cells. Rhabdoid cells could be an important diagnostic clue. Immunohistochemical staining for SMARCA4 (BRG1) should be performed in gastric cancers with solid architecture and undifferentiated components, especially those with rhabdoid morphology.

*SMARCA4* mutations occurred in 8% (20/258) of gastric cancers in the TCGA study and 10% (5/50) of gastric cancers in Takeshima’s study [27, 28]. The incidence of *SMARCA4* mutations was higher in SMARCA4-lost GC than in SMARCA4-reduced cases (6/13 versus 1/14) [29]. Aberrant SMARCA4 protein expression was more common in solid-type poorly differentiated adenocarcinomas (49%, 25/51) than in nonsolid-type poorly differentiated adenocarcinomas (7.5%, 3/40) [12], this report did not describe the complete or partial loss of SMARCA4 protein expression. The relationship between *SMARCA4* gene alteration and SMARCA4 immunoexpression remains elusive, and the genetic underlying cause merits further investigation. Huang, S. C. et al. reported [29] that 2% (27/1199) with altered SMARCA4 expression were identified, exhibiting completely lost (*N* = 6), reduced (*N* = 9) or heterogeneous (*N* = 12) patterns, and seven *SMARCA4* mutations were identified in seven cases, including three SMARCA4-lost gastric cancers harboring *SMARCA4* gene alterations with p. K1091X, p. Y1076X, p. G775S, one SMARCA4-reduced gastric cancer harboring *SMARCA4* gene alterations p. L796F, and three SMARCA4-heterogeneous gastric cancers harboring *SMARCA4* gene alteration patterns with p. K540X, p. R397X and p.R1135W. Another study reported a rare synchronous malignant gastrointestinal neuroectodermal tumor and SMARCA4-deficient undifferentiated carcinoma in the small intestine that showed complete SMARCA4 deficiency with *SMARCA4* frame-shift mutation [c.4882_4886dup(p.Lys1630fs) [30]. In the present study, four cases had complete loss of SMARCA4 (BRG1) immunoexpression. Cases 1 and 3 had *SMARCA4* gene alterations (deletion mutation of *SMARCA4* in case 1 and translocation of *LDLR and SMARCA4* in case 3). *SMARCA4* is located approximately 36 kb from the *LDL-receptor (LDLR)* gene [31]. *SMARCA4/LDLR* has genome-wide significance across phenotypes of statin-induced low-density lipoprotein cholesterol (LDL-C) change [32]. The *SMARCA4* locus near the *LDLR* had the strongest negative association with coronary artery disease (CAD) in this high-risk familial hypercholesterolemia (FH) cohort [33]. The fusion of *LDLR and SMARCA4* was first reported in malignant tumors, and resulted in deficient immunoexpression of SMARCA4. In routine pathology practice, immunohistochemical staining for SMARCA4 (BRG1) specifically detecting *SMARCA4* gene alteration has emerged as a highly useful adjunct tool.

Gastric SWI/SNF complex–deficient tumors may have similar histopathological features with undifferentiated or poorly differentiated morphology [7]. Agaimy et al. reported that most gastric undifferentiated/rhabdoid carcinoma cases (12/13) in the GI tract lack expression of at least 1 of the 4 switch/sucrose nonfermenting (SWI/SNF) complex subunits (SMARCB1, SMARCA2, SMARCA4, and ARID1A) [6]. In 477 adenocarcinomas of the stomach and gastroesophageal junction, 32% of cases demonstrated aberrant expression of the SWI/SNF complex, and SWI/SNF aberration emerged as an independent negative prognostic factor for overall survival [14]. However, *SMARCB1, SMARCA2* and *ARID1A* were not detected by NGS in the present two cases. D-MMR is a good independent prognostic factor in advanced gastric cancer [34]. The SWI/SNF mutations are enriched in microsatellite instability (MSI) genotype [14]. *SMARCA4* mutation was detected in 48.84% of 43 cases with d-MMR GC patients by NGS [35]. SWI/SNF loss is superimposed on mismatch repair deficiency in a subset of cases [6]. Sasaki T et al. demonstrated that 52.0% (13/25) of solid-type poorly differentiated adenocarcinoma had deficient mismatch repair d-MMR [12]. In the present study, 25% (1/4) of cases had d-MMR.

A group of tumors with similar morphologic features should be excluded before diagnosing gastric SMARCA4-UT. The differential diagnosis for gastric SMARCA4-UT includes gastric undifferentiated or poorly differentiated carcinoma with SMARCA4 deficiency, neuroendocrine carcinoma (NEC), EBV-associated carcinoma with lymphoid stroma, lymphomas (including anaplastic large cell lymphoma), melanoma, germ cell neoplasms, NUT-midline carcinoma and so on. Gastric SMARCA4-deficient carcinoma could be distinguished from gastric SMARCA4-UT by gland architecture, cellular cohesion, and diffuse strong keratin expression. Decreased expression of PCK was observed in 58.6% (17/29) of gastric SMARCA4-deficient undifferentiated carcinomas [13]. SALL4 and CD34 were positivity in some gastric SMARCA4-UTs. NEC often diffusely expresses the neuroendocrine markers CgA, Syn and CD56, and tumor cells express epithelial markers, which are helpful for the diagnosis of NEC with large-cell and/or rhabdoid features. The neuroendocrine marker Syn was positively expressed in cases 1, 2 and 3, however, none of the cases exhibited co-expression of other neuroendocrine markers CgA and CD56. Foci of abrupt squamous differentiation can often be identified in NUT-midline carcinoma, and tumor cells can be shown to harbor *BRD-NUT* fusions and NUT-positive expression.

Further analysis showed that patients with SMARCA4-altered GC did not benefit from chemotherapy in stages II and III (*p* = 0.623 and 0.678). In patients with stage III disease who received chemotherapy, SMARCA4-altered GC remained a significant unfavorable prognostic factor (median survival 14 versus 26 months, *p* = 0.002) [29]. Another study demonstrated that the response to preoperative chemotherapy of SWI/SNF-aberrant gastric carcinomas were TRG2 (22%, 8/41) and TRG3 (78%, 32/41) [11]. SMARCA4-altered gastric cancers may do not benefit from chemotherapy and had poor outcomes. Two patients localized at the gastroesophageal junction received neoadjuvant chemotherapy and showed no response (TRG3), showing very adverse clinical characteristics and poor survival [11]. BRG1-associated expression of 9–27 and IFI-27 is involved in cisplatin resistance in gastric cancer cells [36]. In the present study, case 1 received chemotherapy and a PD-1 inhibitor before surgical treatment, and the response to conventional chemotherapy was ineffective. The tumor showed TRG3 in response to neoadjuvant therapy. Future treatments with target agents such as inhibitors against enhancer of zeste homolog 2 (EZH2) or histone deactylase, may prove even more effective [37, 38].

## Conclusions

The present four rare gastric SMARCA4-UTs were aggressive malignancies, occurred in male patients, with advanced stages. These tumors showed solid architecture with undifferentiated morphology. Immunohistochemical staining was negative expression of epithelial markers and complete loss of SMARCA4. Further studies with larger sample size are needed.

## Methods

### Case selection

The data of four cases of gastric SMARCA4-UT were reviewed from the database of the Department of Pathology, West China Hospital, Sichuan University between 2019 and 2022. We retrospectively recorded clinicopathological and demographic characteristics. Clinical and radiographic features were obtained from patients’ medical records and follow-up.

### H&E and immunohistochemical staining

H&E and immunohistochemical staining was performed on 4-µm-thick unstained sections cut from representative formalin-fixed paraffin-embedded blocks. Immunohistochemistry was performed by the avidin–biotin-peroxidase complex technique. Antigen retrieval and staining were performed using standardized automated protocols in the presence of appropriate controls. SMARCA4 (anti-BRG1 antibody, 1:200 dilution, clone EPNCIR111A; Abcam, Cambridge, MA) was performed. Pancytokeratin(PCK) (clone AE1/AE3, ZSGB-BIO), epithelial membrane antigen (EMA)(clone GP1.4, ZSGB-BIO), CK20(clone EP23, ZSGB-BIO), CK7(clone EP16, ZSGB-BIO), CK8/18(clone 5D3, MXB), CD34(clone EP88, ZSGB-BIO), SMARCB1(INI1) (clone 25, ZSGB-BIO), p53(clone D0-7, ZSGB-BIO), synaptophysin (Syn) (clone EP158, ZSGB-BIO), chromogranin A(CgA) (clone LK2H10, ZSGB-BIO), CD56(clone UMAB83, ZSGB-BIO), S100(clone 4C4.9, MXB), HMB45(clone HMB45, MXB), CD117(clone YR145, MXB), DOG1 (clone SP31, MXB), CD99(clone EP8, ZSGB-BIO), LCA(clone 2B11&PD7/26, ZSGB-BIO), Desmin(clone MX046, MXB), WT-1(clone EP122, ZSGB-BIO)), Ki67(clone MIB-1, ZSGB-BIO), MLH1(clone ES05, ZSGB-BIO), MSH2 (clone RED2, ZSGB-BIO), MSH6(clone EP49, ZSGB-BIO)), PMS2 (clone EP51, ZSGB-BIO), and nuclear protein in testis (NUT) (clone B1, ZSGB-BIO) and SALL4(clone 6E3, ZSGB-BIO) were performed. The staining was determined by 2 independent pathologists.

### In situ hybridization of Epstein‒Barr virus-encoded small RNA (EBER)

We stained 4 μm thick sections for in situ hybridization to examine the Epstein‒Barr virus (EBV) infection status. The EBER probe was detected using the PNA ISH Detection Kit (Dako).

### Next generation sequencing (NGS)

Cases 1 and 3 with abundant tumor tissue and sufficient well-preserved nucleic acids for sequencing were submitted for next-generation sequencing (NGS). DNA was extracted from paraffin-embedded tissue blocks using Qiagen AllPrep kits (Qiagen, German). Custom probes were used to produce an enriched library containing all exons from 1021 cancer-related genes by Geneplus Technology (case 1) and 425 cancer-related genes panel by Geneseeq Technology (case 3), and sequencing was performed on the NextSeq 550 (Illumina, San Diego, CA) with a median target exon coverage of 900×. Variants were reviewed using Integrated Genomics Viewer (Broad Institute, Cambridge, MA), and somatic variants were identified on the basis of variant allele frequencies and databases including gnomAD and dbSNP.

## Data Availability

The original contributions presented in the study are included in the article. Further inquiries can be directed to the corresponding author.

## Declarations

### Headings

Gastric SMARCA4-deficient undifferentiated tumor (SMARCA4-UT) is a rare entity of gastric cancer that is typically negative expression of epithelial markers and deficient expression of SMARCA4. Herein, we report four rare cases of gastric SMARCA4-UTs.

## Abbreviations

SWI/SNF: SWItch/Sucrose Non-Fermentable
BRG1: Brahma-related gene 1
SMARCA4-UT: SMARCA4-deficient undifferentiated tumor
CT: Computed tomography
PCK: Pancytokeratin
EMA: Epithelial membrane antigen
Syn: Synaptophysin
CgA: Chromogranin A
EBER: Epstein‒Barr virus-encoded small RNA
TNM: Tumor-node-metastasis
NGS: Next-generation sequencing
TRG: Tumor regression grade
